# Evolution of Surgical Management of Hemorrhoidal Disease: An Historical Overview

**DOI:** 10.3389/fsurg.2021.727059

**Published:** 2021-08-30

**Authors:** Francesco Pata, Gaetano Gallo, Gianluca Pellino, Vincenzo Vigorita, Mauro Podda, Salomone Di Saverio, Giancarlo D'Ambrosio, Giuseppe Sammarco

**Affiliations:** ^1^General surgery Unit, Nicola Giannettasio Hospital, Corigliano-Rossano, Italy; ^2^La Sapienza University, Rome, Italy; ^3^Department of Medical and Surgical Sciences, University of Catanzaro, Catanzaro, Italy; ^4^Department of Advanced Medical and Surgical Science, Università degli Studi della Campania “Luigi Vanvitelli”, Naples, Italy; ^5^Colorectal Surgery, Vall d'Hebron University Hospital, Barcelona, Spain; ^6^Coloproctology Unit and General and Digestive Surgery Unit, Vigo University Hospital Complex, Vigo, Spain; ^7^Department of emergency surgery, Cagliari University Hospital “Policlinico D. Casula”, Cagliari, Italy; ^8^Department of General Surgery, University Hospital of Varese, ASST Sette Laghi, University of Insubria, Varese, Italy; ^9^Department of General Surgery, Surgical Specialties and Organ Transplantation, La Sapienza University, Rome, Italy; ^10^Department of Health Science, University of Catanzaro, Catanzaro, Italy

**Keywords:** haemorroidal disease, history, surgical therapies, haemorroidectomy, haemorroids, hemorrhoids, hemorrhoidal, surgery

## Abstract

Hemorrhoidal disease (HD) is the symptomatic enlargement and/or distal displacement of the normal hemorrhoidal cushions and is one of the most frequent diseases in colorectal surgery. Several surgical or office-based therapies are currently available, with the aim of being a more tailored approach. This article aimed to elucidate the historical evolution of surgical therapy for HD from ancient times, highlighting the crucial steps, controversies, and pioneers in the field. In contrast with the previous literature on the topic that is often updated to the 1990s, with the introduction of stapled hemorrhoidopexy and transanal hemorrhoidal dearterialization, this article describes all new surgical and office-based treatments introduced in the first 20 years of the 2000s.

## Introduction

Hemorrhoidal disease (HD) is the symptomatic enlargement and/or distal displacement of the normal anal cushions called hemorrhoids (1) and is the most common anorectal disorder (2, 3). It has been reported that more than 50% of people present at least one episode of symptomatic hemorrhoids during their life (4), and a significant proportion will undergo surgery if unresponsive to conservative treatment. Over the past 100 years, many advances have been made in the surgical approach to HD. New surgical and office-based procedures have been developed to reduce postoperative pain and complications and improve long-term efficacy. The aim of this article is to describe the history of surgical therapy for HD, highlighting the crucial steps and major contributors in this field. In contrast with previous literature that is often updated up to stapled hemorrhoidopexy as the most recently available procedure, all new techniques introduced in clinical practice after the year 2000 are reported.

The introduction of anesthesia and antisepsis in the middle of 19th century created a watershed between a ***pre-modern era***, in which any surgical therapy of HD was a gory experience, often based on empirical rather than theoretical principles, a very poor understanding of human anatomy, and a high risk of mortality and complications, and a ***modern era***, in which several surgical techniques were finally standardized and developed in safe conditions to achieve a curative effect with low risk of complications.

## Pre-Modern ERA

Old Testament and Egyptian scriptures are the first documents that mentioned anal symptoms suggestive of hemorrhoids and their therapy (5). However, it is impossible to confirm whether the nature of the anal disease and the symptoms described are actually related to hemorrhoids or might have referred to other anorectal diseases such as condylomas or syphilis. “*Before Hippocrates” time 5th Century BC any disease in or around the anus was called hemorrhoids*” (6).

In the Old Testament, God punishes Philistines with “*emerods*” (1 Samuel 5:6), while in Deuteronomy (27:28), Moses warn the Israelites that, in case of breaking the law of God, “*The LORD will smite thee with the botch of Egypt, and with the emerods, and with the scab, and with the itch*.” Although the term “emerods” was first reported and popularized by the King James Bible (1611), the actual word used in the Hebrew text was *techorim*, which might have been better translated as “*tumor” or “round shaped tumor-like appendage or protrusion from anus”* (7, 8), making it impossible to unequivocally identify this as hemorrhoids.

Edwin Smith Papyrus (1700 BC) and the Ebers Papyrus (1500 BC) recommend astringent lotions containing honey, myrrh, flour, ibex fat, and sweet beer for anal symptoms that are strongly suggestive of symptomatic hemorrhoids (9, 10). However, no surgical therapy is reported.

***Hippocrates*** (460–375 BC), the father of Medicine, was the first author to propose a surgical therapy for symptomatic hemorrhoids. Hippocrates believed that hemorrhoids resulted from an excess of bile or phlegm in the body and that their bleeding was somewhat beneficial, preventing other diseases such as pleuritis or leprosy. However, in other writings, he seems to contradict himself, proposing surgery for hemorrhoids, the principles of which are still valid today: ligation, excision, or cauterization. In the *Treatise on Hemorrhoids*, he suggests treating hemorrhoids by “*transfixing them with a needle and tying them with very thick and woolen thread*” (11), and in the “On Hemorrhoids” text, he advocates hemorrhoids excision and describes a rectal speculum similar to the Eisenhammer retractor (11). Cauterization is also proposed: “*Having on the preceding day first purged the man with medicine, on the day of the operation apply the cautery*. […] *Having laid him on his back, and placed a pillow below the breech, force out the anus as much as possible with the fingers […] And burn so as to leave none of the hemorrhoids unburnt. […] When the cautery is applied the patient's head and hands should be held so that he may not stir, but he himself should cry out, for this will make the rectum project the more. When you have performed the burning, boil lentils and tares, finely triturated in water, and apply as a cataplasm for 5 or 6 days*.” (12).

Roman medicine basically resumed the Egyptian and Greek traditions without any innovative contribution. ***Celsus*** (1st century AD), in the seven books of “*Re Medica*,” recommended either the ligation of hemorrhoids by flax followed by the excision of the ligated nodule, or the excision alone followed by a transfixed stitch in case of large hemorrhoidal nodules (13). ***Galen*** (130 −200 AD) suggested a conservative management based on laxatives, leeches, and ointment (10), proposing ligation by a tight thread as the only surgical option.

During the Middle Ages until the 18th century, there have not been great advances in the management of hemorrhoids. The principles and operations described by classic authors were pedantically reported by Arabic and European authors. The high mortality and complications, as well as the frequent practice of operations by charlatans and barber surgeons, discouraged surgery.

***Herny de Mondeville*** (1260–1320), one of the most influential surgeons of his age, warned about operating hemorrhoids (10). ***Lorenz Heister***, in the book *Chirurgie* (1739), described the “*method of the ancient too cruel, and often perniciosus*” (14) and ***Hugues Ravaton***, in the “Pratique moderne de la chirurgie” (1776), judged that the remedies proposed until then “*by the masters of the Art worked out very poorly*” (6).

Don Juan of Austria, the hero of the battle of Lepanto (1571), died in 1578 for uncontrolled bleeding 4 hours after an operation for hemorrhoids (15).

**Figure 1:**
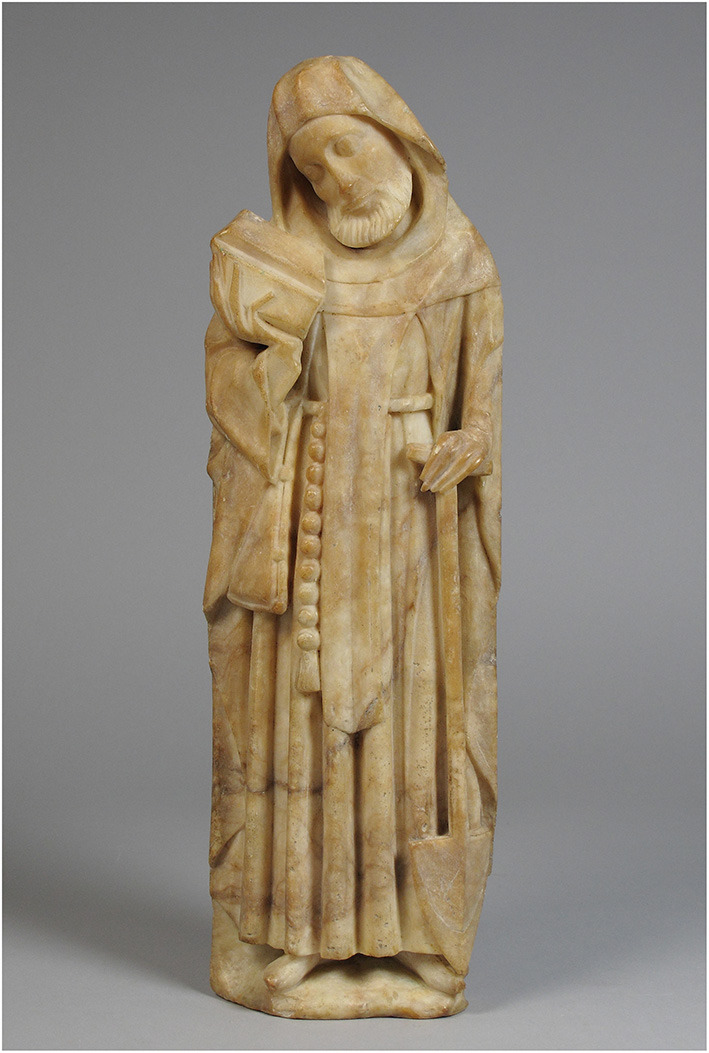
Saint Fiacre, patron saint for hemorrhoid suffers, depicted in a sculpture of mid-15th century (from MET, Metropolitan Museum of Art, New York, USA. https://www.metmuseum.org/).

These poor surgical outcomes spread skepticism about the surgery and encouraged “unconventional” attempts. In the Middle Ages, Saint Fiacre (Figure 1), already the patron of gardeners, became the patron saint of hemorrhoids, from which the “Illness of St. Fiacre” was used as a polite term to indicate the disease. For centuries, the monastery of St. Fiacre (France) was a place of pilgrimage, in which sufferers of hemorrhoids were used to sit on a stone considered able to cure the disease (16, 17).

The 18th century marked some advancement in the understanding of HD, breaking some dogmas of the Hippocratic tradition. ***Giovanni Battista Morgagni*** (1682–1771) attributed the etiology of hemorrhoids to the upright posture of humans and to a hereditary predisposition, recognizing the absence of a valve in the rectal veins as a contributing factor (18). ***George*** ***Ernst Stahl*** (1660–1734), an eminent German Professor of Medicine, defined hemorrhoids as venous reservoirs, whose bleeding was somewhat beneficial as an expression of surplus of blood (19).

It is worth mentioning that the defeat of Napoleon at Waterloo on June 18, 1815 was partially attributed to an episode of presumably thrombosed hemorrhoids that likely affected his performance on the crucial day of the battle (20).

Indeed, the fear of uncontrolled bleeding and lethal sepsis with inability to relieve pain represented significant obstacles to the surgical therapy for hemorrhoids until the mid-19th century, when anesthesia and antisepsis inaugurated scientific surgery.

## Modern ERA

### 19th Century

Three main surgical trends characterized the 19th century: anal stretching, excision, and sclerotherapy.

In 1835, ***Frederick Salmon*** founded in London the “*Benevolent Dispensary for the Relief of the Poor Afflicted with Fistula, Piles and other Diseases of the Rectum and Lower Intestines*,” which was later moved to a larger premise in London and named “*St Mark's Hospital for Fistula and other Diseases of the Rectum*” (officially opened in 1853) (21). This was the first institution dedicated to the treatment of anorectal disease ^1^.

Salmon first proposed anal stretching to treat hemorrhoids (22), and then, in the second part of his career, he performed a personal technique for hemorrhoid excision, laying the foundation for open hemorrhoidectomy that was popularized by Milligan and Morgan in the 20th century. Salmon described that internal hemorrhoids were supplied by the superior hemorrhoidal artery, and, as described by Allingham in 1888, he performed a combined technique of excision of the hemorrhoidal nodules by incising perianal skin and ligation of the pedicle above the dental line to reduce pain (23). However, postoperative strictures were common (24).

In 1855, ***Aristide Auguste Stanislas Verneuil*** (1823–1895) suggested that anal dilatation (also called “*rectal bouginage*”) was beneficial in the treatment of hemorrhoids, because increased anal tone was considered the cause of HD. The technique gained popularity over the 19th century (25), especially in France and in the United States, originally as a two-finger dilatation technique, which was later replaced by the use of anal dilators, such as the Manx dilators, introduced by Percy Lockhart-Mummery (26, 27). They were easy to introduce, and not slipping out owing to its shape. In 1969, ***Lord*** popularized again the technique (28). According to his theory, the aim was to stretch the fibrotic bands in the internal anal sphincter, causing obstruction and venous engorgement on the basis of the hemorrhoids. This technique was still advocated in the 1980s (29). Currently, this technique has been abandoned, as it may cause injuries to the internal anal sphincter.

In 1882, ***Walter Whitehead*** (1840–1913) proposed a radical approach for the excision of circumferential hemorrhoids (30). He proposed “*the excision of the complete ring of pile-bearing mucous membrane*” by a circular incision at the level of mucocutaneous border (clearly corresponding to the dentate line), without leaving any mucocutaneous bridge, thus removing the entire segment of dilated hemorrhoidal cushions and the overlying mucosa, suturing the proximal end to the skin below.

In 1887, ***Whitehead*** published the first 300 cases that underwent his technique, which was slightly modified compared with the original description, and reported no cases of stenosis or ectropion (31). He highlighted that no skin sacrifices would have occurred. The operation gained wide popularity in the following decades, which gradually decreased in the 20th century due to the high rate of complications reported, such as anal stenosis, incontinence, or persistent soiling due to mucosal ectropion and deformity (also called “Whitehead's anus”) (32). In 1924, J. Lockhart-Mummery declared that the death knell of Whitehead operations had been sounded during the London meeting of the American Proctologic Society (33). Nevertheless, Whitehead operation is still performed at some centers for circumferential 4-degree hemorrhoids with acceptable results (34–37). Probably, the poor results of this operation are often the consequence of an incorrect technique, such as excision of the skin or misidentification of the mucocutaneous junction, corresponding to the dentate line, that some surgical texts between the mid-1800s and the first half of the 1900s erroneously identified with the white line of Hilton or the intersphincteric line, 1.3 cm on average distally located (38). “*Mistaking the white line of Hilton for the mucocutaneous junction would mean the difference between good results and mucosal ectropion or a stricture*” (39).

Around the 1860s, the injection of sclerosing agents was introduced in clinical practice, although the technique was already in use by quacks, known as “healers of hemorrhoids,” in the United Kingdom and in the USA (6, 9, 10). In 1869, ***James Morgan***, a surgeon in Dublin, first described sclerotherapy using iron sulfate (40). Ten years later, in 1879, ***Andrew Edmunds***, at the Chicago Medical Society meeting, reported 3,000 cases of sclerotherapy for hemorrhoids, mainly by carbolic acid and olive oil, with nine cases of death, 23 cases of major complications such as abscess, dangerous postoperative bleeding, and embolism to the liver, and 25% of severe pain (41). In 1888, ***Swinford Edwards***, from St. Mark, reported the results of 38 patients treated with sclerotherapy with carbolic acid over a 2-year period, with only one case of recurrence (42).

### 20th Century

**Figure 2:**
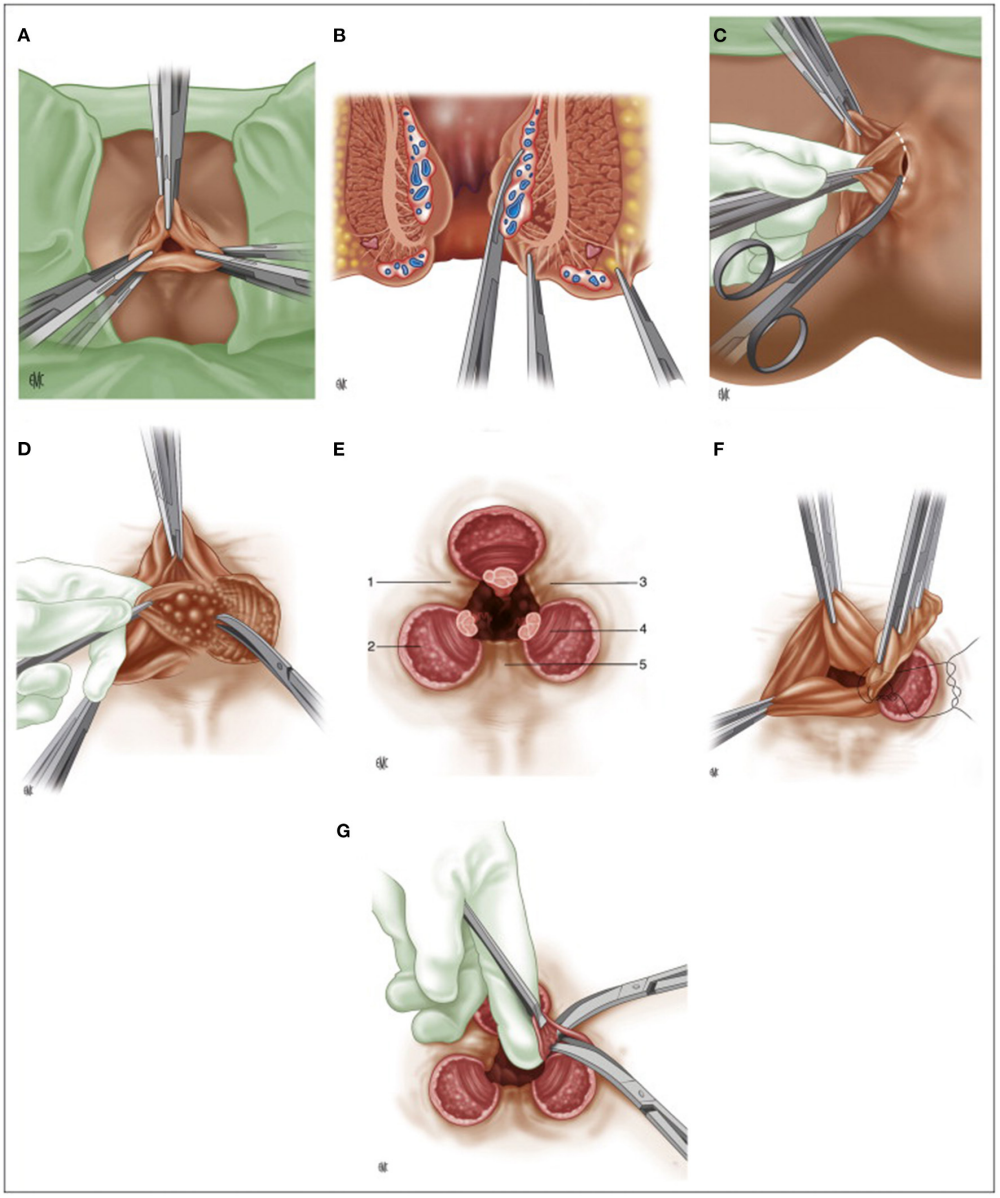
**(A)** Milligan-Morgan hemorrhoidectomy, position of the three sets of clamps; **(B)** Placement of three clamps on the hemorrhoidal group, frontal view; **(C)** Dissection of the left hemorrhoidal group; **(D)** Exposure of the internal sphincter after division of Parks' ligament; **(E)** Final post-operative appearance. 1: left anterior muco-cutaneous bridge. 2: internal anal sphincter. 3: posterior muco-cutaneous bridge. 4: sub-cutaneous fibers of the external anal sphincter. 5: right anterior muco-cutaneous bridge; **(F)** Suture ligature of the hemorrhoidal pedicle; **(G)** Cleaning up the muco-cutaneous bridges. (Reproduced from Moult HP, Aubert M, De Parades V. Classical treatment of hemorrhoids. *J Visc Surg*. (2015) 152:S3–9. Copyright © 2014 Elsevier Masson SAS. All rights reserved).

The first part of the 20th century was characterized by the affirmation of open hemorrhoidectomy as the gold standard treatment for HD (43). Salmon's operation was slightly modified by several authors, such as ***Miles*** (1919) or ***Lockhart-Mummery*** (1923), but in 1937, ***Edward Campbell Milligan*** (1886–1972) and ***Clifford Naughton*** ***Morgan*** (1901–1986), from St. Mark's hospital, standardized the version, whose principles, for the most part, are still valid nowadays (Figure 2): V-shaped incision of the skin, preservation of mucocutaneous bridges to avoid stenosis, meticulous identification of the anatomical canal anatomy and ligation of the hemorrhoidal pedicle, which contains the mucosa, submucosa, the terminal branch of the superior hemorrhoidal artery and vein, and a portion of the anal internal sphincter that in the authors' intentions was necessary to reduce the upward tension and the risk of stenosis (44–46).

In the 1920s, 5% phenol oil became the most used agent in sclerotherapy, although other agents, such as urethane, nitric acid, iodine, alum, or quinine, were reported (47).

**Figure 3:**
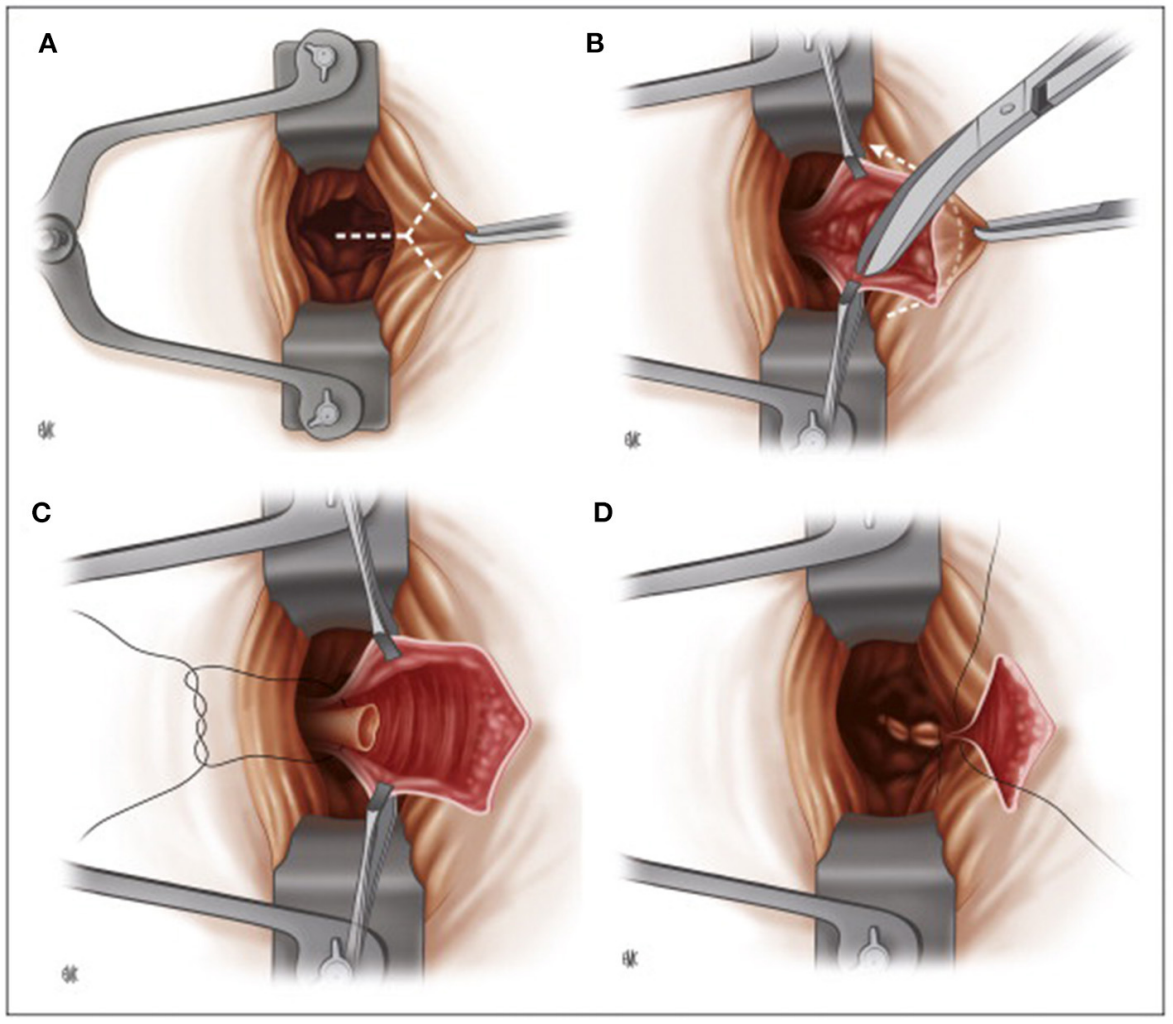
Parks submucosal hemorrhoidectomy. **(A)** Placement of the retractor, intracanular incision. **(B)** Submucosal hemorrhoidectomy. **(C)** Ligation of the pedicle. **(D)** Suture closure of the mucosa of the anal canal (Reproduced from Moult HP, Aubert M, De Parades V. Classical treatment of hemorrhoids. *J Visc Surg*. (2015) 152:S3–9. Copyright © 2014 Elsevier Masson SAS. All rights reserved).

In the 1950s, Sir ***Alan Guyatt Parks*** (1920–1982), from St. Mark's hospital, introduced the submucosal hemorrhoidectomy (Figure 3), publishing the first article in 1956 (48). He considered the Milligan-Morgan technique suboptimal because of excessive sacrifice of the rectal mucosa (with the risk of stenosis) and due to the pedicle ligation in a sensitive area of the anoderm, resulting in excessive postoperative pain. To overcome these issues, he then proposed a mucosal-sparing technique with high ligation of the hemorrhoidal pedicle in an insensitive area of the rectum (49). However, the idea was not entirely original. In 1774, ***J.C. Petit*** had already proposed the treatment of hemorrhoids by a vertical incision at the hemorrhoidal level, removing the submucosal tissue underneath and ligating the pedicle before re-suturing the flaps created (50).

***Parks*** described an inverted Y-incision 3–5 cm starting from the mucocutaneous junction between the mucosa of the upper canal and the anorectal junction. The hemorrhoidal tissue is completely freed from the mucosa on each side and from the muscle plane below. The pedicle was ligated an inch above the mucocutaneous junction using a transfixed 0 chromic catgut stitch. The mucosal flaps were then sutured, and a small skin area was left open to prevent skin tags and allow drainage. No tube or dressing was placed transanally (45, 48).

Submucosal hemorrhoidectomy is technically challenging and time-consuming, with a risk of significant loss of bleeding and fecal incontinence due to the long-lasting application of the Parks self-retractor (50). Therefore, due to the satisfying results of the Milligan-Morgan operation, the technique never became popular. However, it is still performed, although with some changes, for 4-degree hemorrhoids (48) with gratifying outcomes in two randomized controlled trials comparing the operation to the Milligan-Morgan technique (51, 52).

**Figure 4:**
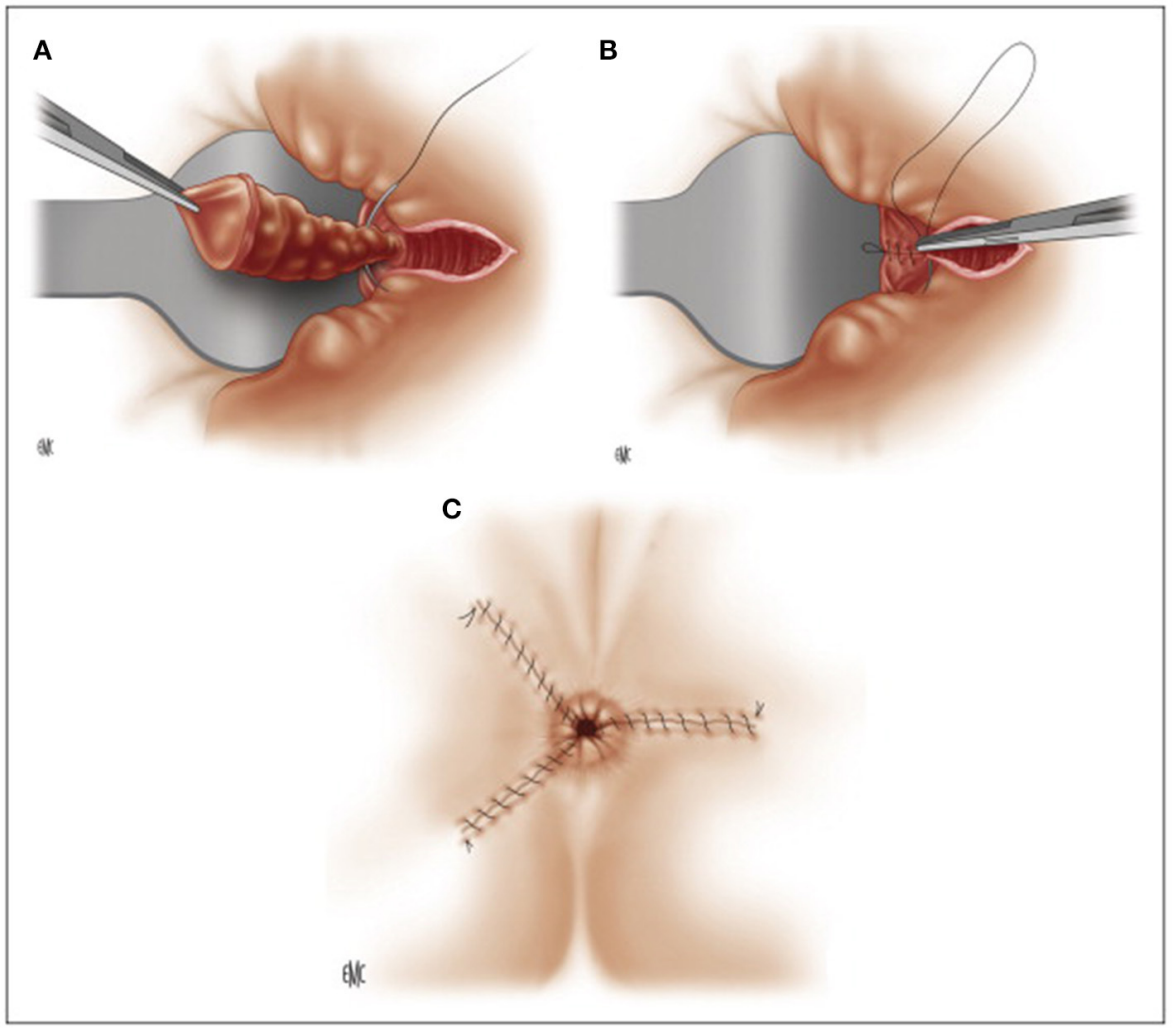
Ferguson hemorrhoidectomy. **(A)** Ligation of the pedicle after dissection. **(B)** Running muco-cutaneous closure. **(C)** Post-operative appearance. (Reproduced from Moult HP, Aubert M, De Parades V. Classical treatment of hemorrhoids. *J Visc Surg*. (2015) 152:S3–9. Copyright © 2014 Elsevier Masson SAS. All rights reserved).

In 1955, ***James A. Ferguson*** described closed hemorrhoidectomy (Figure 4), currently the most popular technique in the USA, with the aim of reducing postoperative pain and bleeding (53). The operation is performed in a similar way as the Milligan-Morgan operation, preserving much mucosa and closing the margins of all wounds by locking stitches, proximally secured with the suture of the pedicle not cut after ligation (45). Some evidence suggests that closed hemorrhoidectomy may have better outcomes, such as reduced postoperative pain, lower risk of postoperative bleeding, and faster wound healing than the Milligan-Morgan operation (54–56).

It is worth mentioning that several surgeons often perform these techniques with personal modifications and/or along with anal stretching or anal sphincterotomy, making it difficult to make any comparison, and explaining some great difference in terms of reported complication rates.

In 1963, ***James Barron*** described rubber banding ligation, an office-based procedure for early stage hemorrhoids, reporting only four cases of bleeding among 200 treated patients (57). He was inspired by ***Paul C. Blaisdell***, who described the application of rubber bands for hemorrhoids by an umbilical cord ligator in 1958 (58). Barron introduced the homonym ligator (Baron ligator), describing technical steps that are still valid today.

Other office-based treatments have been introduced in clinical practice such as cryotherapy (1969) (59) and infrared coagulation (1977) (60), although with less fortune than rubber banding ligation and sclerotherapy.

In the 1990s, pioneer centers started to perform day-case hemorrhoidectomy and ***Sharif*** described the diathermy hemorrhoidectomy, in which the pedicle was not ligated but coagulated by diathermy to reduce postoperative pain, which was often attributed to the ligation of the pedicle (61). He presented the short-term outcomes in 72 patients, reporting two cases of postoperative hemorrhage and no anal stenosis at 6-weeks follow-up (61).

The 20th century ended with two new techniques aimed at treating HD by reducing postoperative pain and minimizing unnecessary sacrifice of hemorrhoids, whose functional role in the sensitivity of the anal canal and in the continence was increasingly recognized.

**Figure 5:**
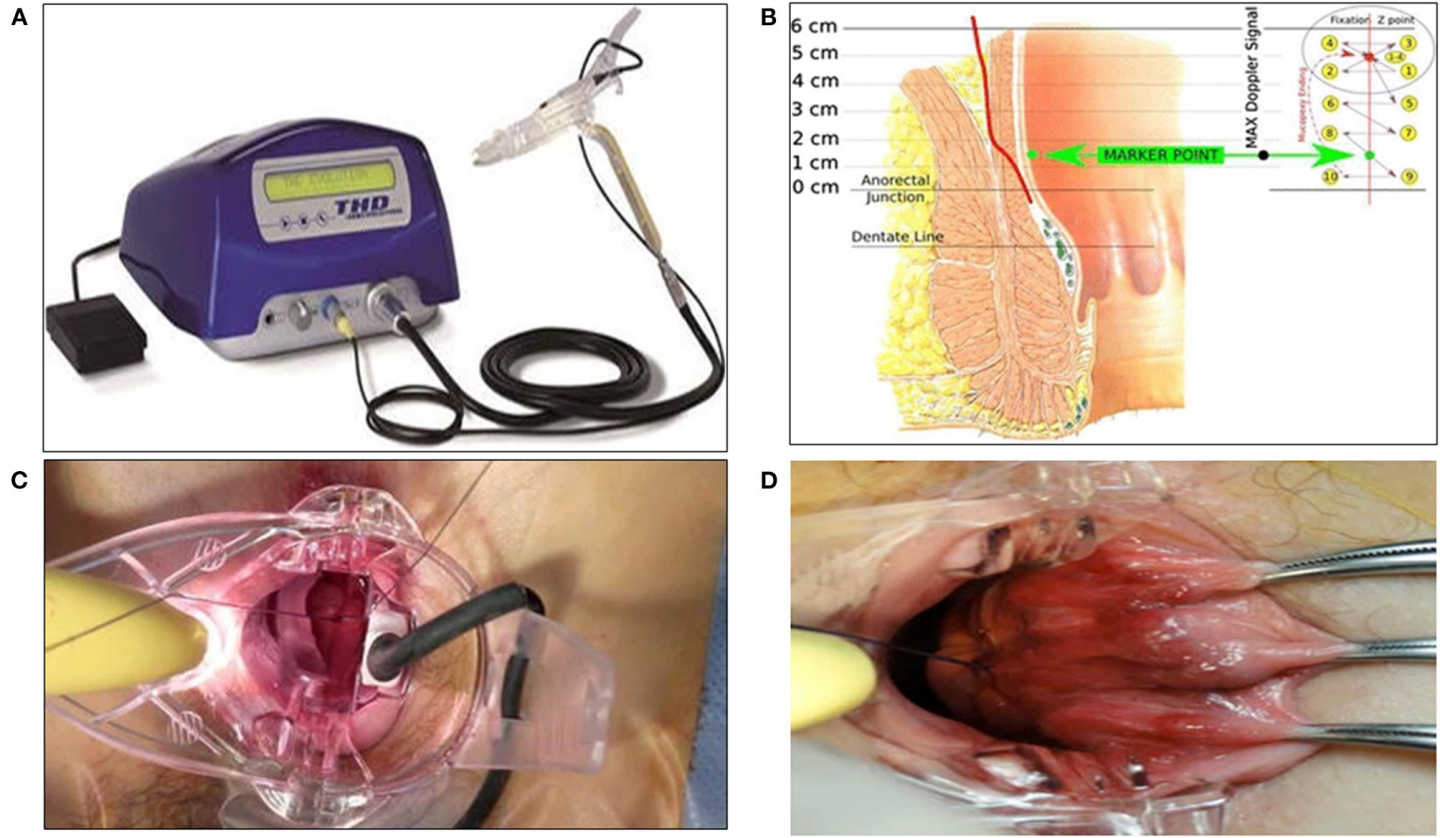
THD Doppler procedure. **(A)** Surgical instruments specifically designed for the THD procedure. **(B)** Schema of the anatomical course of a hemorrhoidal artery and mucopexy fixation point and continuous suture. **(C)** Suture of a hemorrhoidal artery during DDD (Distal Doppler-guided dearterialization). **(D)** Mucopexy suture is secured without including the hemorrhoids. (Reproduced from Ratto C. THD Doppler procedure for hemorrhoids: the surgical technique. *Tech Coloproctol*. (2014) 18:291–298. Creative Commons Attribution 2.0 International License).

In 1995, ***Morinaga*** described hemorrhoidal artery ligation (HAL) or transanal hemorrhoidal dearterialization (THD) based on Doppler-guided ligation of the terminal branches of the hemorrhoidal arteries by a designated proctoscope associated with a Doppler probe (62). Once identified by Doppler, each terminal arterial branch is ligated using a figure-eight suture. Six ligations are usually necessary. This results in reduced blood supply of the hemorrhoidal plexus, causing atrophy and fibrosis. Currently, this technique is combined with mucopexy or rectoanal repair (RAR), not necessarily using Doppler US, to treat the prolapse and to improve long-term results (Figure 5) (63, 64).

In 1998, ***Antonio Longo*** described stapled hemorrhoidectomy, referred to as the *Longo technique* or stapled hemorrhoidopexy (which seems more appropriate) (65). The operation aims to resect a circular layer of rectal mucosa above the hemorrhoids using a dedicated stapler (initially PPH 1, then PPH 3), reducing the blood flow to the hemorrhoidal plexus and lifting the hemorrhoids in anatomical position, resolving the prolapse. This technique has gained worldwide popularity as a painless technique with excellent short-term results. To increase the long-term results, new devices, such as high-volume staplers, were introduced in the early 2000s (66). The higher long-term recurrences compared to hemorrhoidectomy and the report of serious, albeit rare, complications have reduced the adoption of the technique in many centers (67). Nevertheless, stapled hemorrhoidopexy highlighted two key concepts on which many new surgical techniques for HD are based: avoiding skin excision, as one of the main contributors of postoperative pain, and directing the operation above the dentate line, recognizing the functional role of hemorrhoidal tissue (68).

### 21st Century

In the first 20 years of the 2000s, the surgical treatments for hemorrhoids have been moving in two directions: on one hand, traditional techniques have been modified according to new devices to increase postoperative outcomes; on the other hand, minimally invasive techniques have been developed to reduce postoperative pain, need for hospital admission, and injuries to the structures of the anal canal. Several authors have described open hemorrhoidectomy performed by high-energy devices, such as ultrasound or radiofrequency, with promising results in some series (69, 70).

According to the “vascular theory,” which postulates the blood overflow from the superior hemorrhoidal artery as the main cause of hemorrhoidal disease, and thanks to the positive results of THD, Hemorrhoids Laser Procedure (HeLP) and Emborrhoid were developed as new mini-invasive surgical treatments.

**Figure 6:**
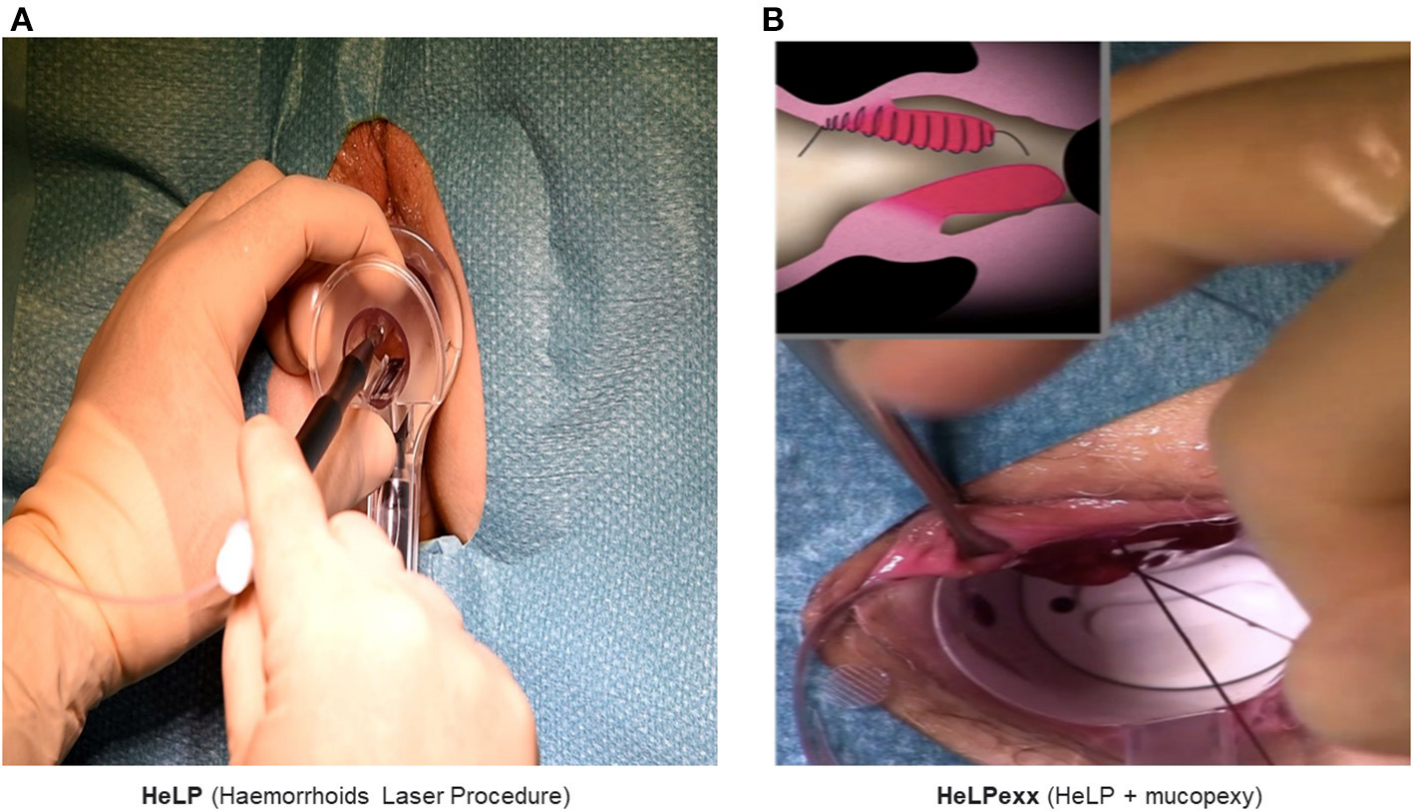
HeLP **(A)** and HeLPexx **(B)** procedures (courtesy of Paolo Giamundo MD, FEBSQ, FRCSE).

HeLP, first described in 2011 by ***Paolo Giamundo*** (71), involves selective closure of the terminal branches of the superior rectal arteries, which were identified by a 20 MHz Doppler probe, 3 cm proximal to the dentate line using a laser optic fiber (Figure 6A). It was initially indicated for 2- or 3-degree hemorrhoids without significant prolapse, although recently, a combination with mucopexy (HeLPexx) has been described (Figure 6B), widening the indications for advanced HD (72).

**Figure 7:**
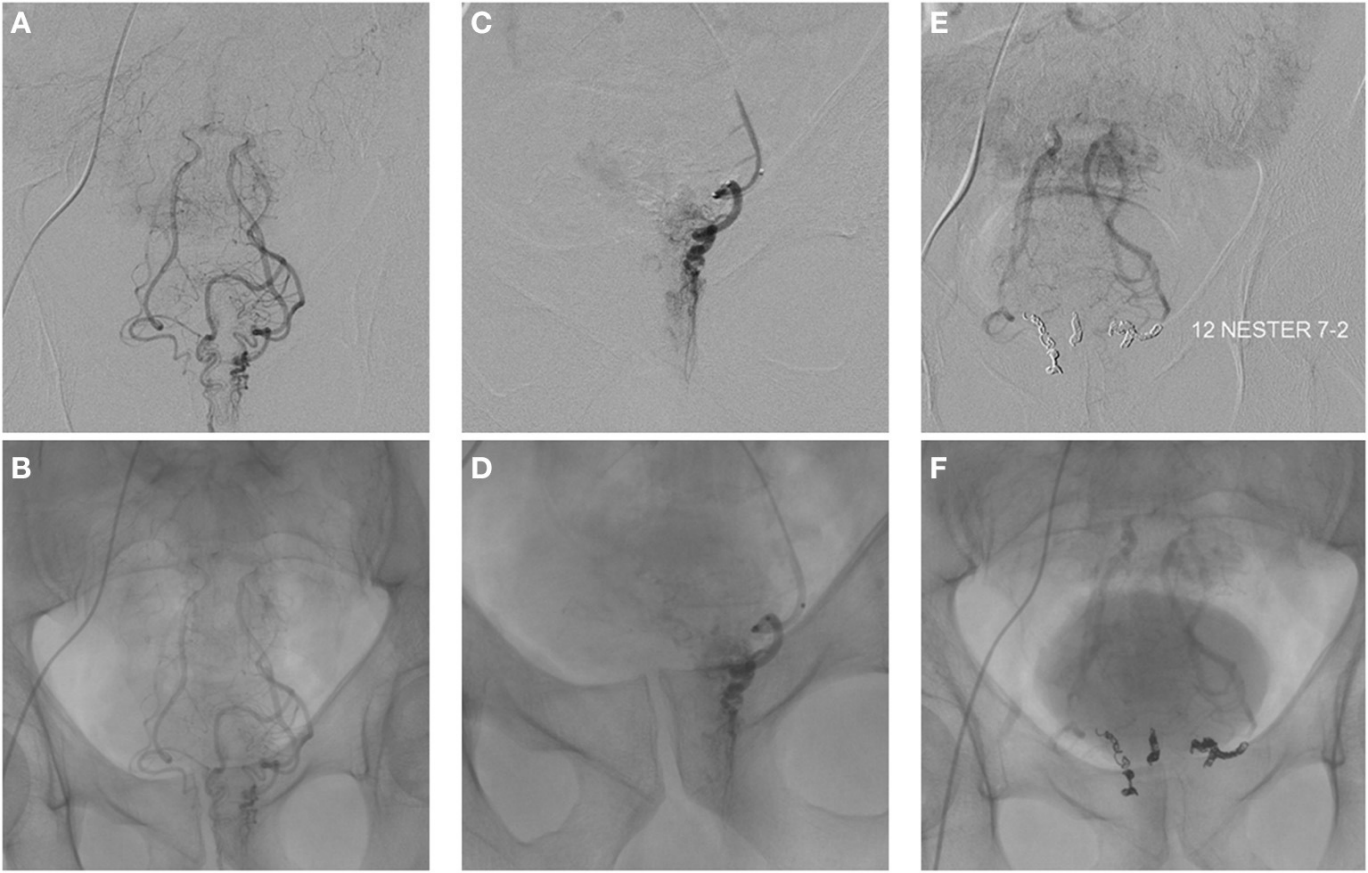
Emborrhoid technique (courtesy of Prof. Vincent Vidal).

**Figure 8:**
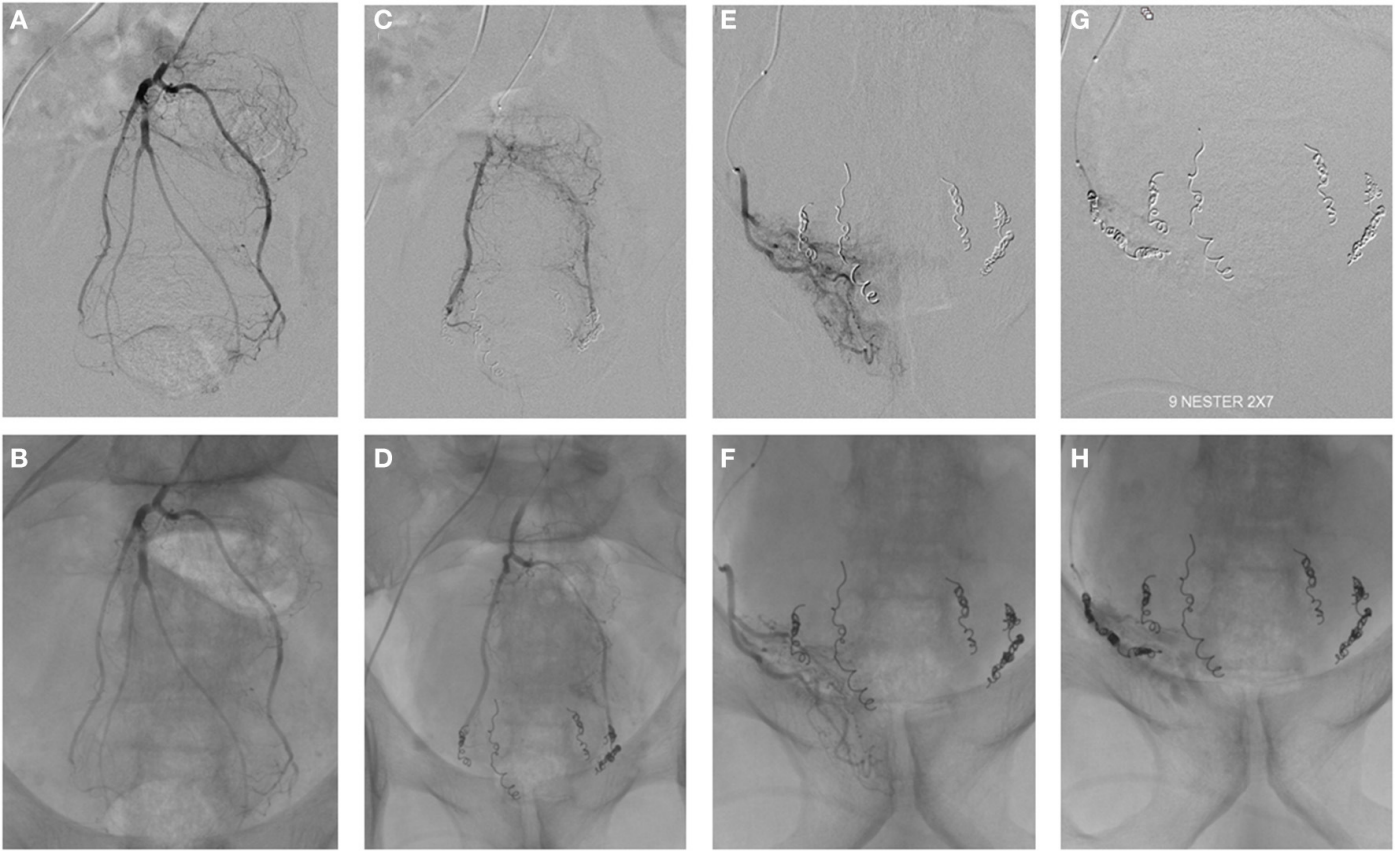
Emborrhoid technique (courtesy of Prof. Vincent Vidal).

In 2014, Emborrhoid, a radiological interventional technique (Figures 7, 8), was described by ***Vincent Vidal*** based on selective embolization of the terminal branches of the superior rectal artery (73, 74). In analogy with the principles of THD, the terminal branches of the superior rectal arteries are occluded by coils placed by the endovascular route. It is generally indicated in patients not fit for surgery, with major/life-threatening bleeding and unresponsive to conservative therapy (72).

In 2007, sclerotherapy received a new impetus with the introduction of 3% polidocanol foam as a sclerosing agent by ***Moser*** (75). Since then, several studies have shown the superiority of foam in terms of effectiveness and reduced complications compared with oil-based agents, inaugurating a new era for sclerotherapy (76–79). However, these studies mainly focused on the treatment of 1-degree HD, while further studies are needed on 2- and 3-degree HD (79, 80).

In 2021, Sclerobanding, a combined technique of sclerotherapy with 3% polidocanol foam and rubber banding ligation, was described by ***Bracchitta*** et al. (81). The aim of the authors is to further increase the results of both techniques, reducing the risk of delayed bleeding and abscess associated with each technique when applied alone.

All these techniques can be performed as an office-based procedure, under or even without local anesthesia, and they are repeatable in cases of recurrence.

The current mainstream surgical management of HD is represented by using office-based procedures and minimally invasive techniques whenever possible: the wide range of options available allows a tailored approach because “no one size fits all options” (82).

## Author Contributions

FP, GG, GP, VV, MP, and SD: drafting and writing. GD'A and GS: revision of the text, adding new contributions. All authors read and approved the final version of the manuscript.

## Conflict of Interest

The authors declare that the research was conducted in the absence of any commercial or financial relationships that could be construed as a potential conflict of interest.

## Publisher's Note

All claims expressed in this article are solely those of the authors and do not necessarily represent those of their affiliated organizations, or those of the publisher, the editors and the reviewers. Any product that may be evaluated in this article, or claim that may be made by its manufacturer, is not guaranteed or endorsed by the publisher.

## Footnotes

^1^Available online at: https://www.stmarkshospitalfoundation.org.uk/about/history/(accessed 09 Jun, 2021).

## Abbreviations

HD: Hemorrhoidal Disease
HAL: hemorrhoidal artery ligation
THD: transanal hemorrhoidal dearterialization
RAR: recto-anal repair
HeLP: Hemorrhoids Laser Procedure.
